# Integration of temperature-driven population model and pest monitoring data to estimate initial conditions and timing of first field invasion: application to the cassava whitefly, *Bemisia tabaci*

**DOI:** 10.1098/rsif.2025.0059

**Published:** 2025-05-07

**Authors:** Frank Thomas Ndjomatchoua, Richard Olaf James Hamilton Stutt, Ritter A Guimapi, Luca Rossini, Christopher A Gilligan

**Affiliations:** ^1^ Department of Plant Sciences, University of Cambridge, Cambridge, United Kingdom; ^2^ Biotechnology and Plant Health Division, Norwegian Institute of Bioeconomy Research, Ås, Norway; ^3^ Service d'Automatique et d'Analyse des Systèmes, Université Libre de Bruxelles, Brussels, Belgium

**Keywords:** insect pest monitoring, simulation model, physiological age, pioneer invader, whitefly

## Abstract

Empirical field data and simulation models are often used separately to monitor and analyse the dynamics of insect pest populations over time. Greater insight may be achieved when field data are used directly to parametrize population dynamic models. In this paper, we use a differential evolution algorithm to integrate mechanistic physiological-based population models and monitoring data to estimate the population density and the physiological age of the first cohort at the start of the field monitoring. We introduce an ad hoc temperature-driven life-cycle model of *Bemisia tabaci* in conjunction with field monitoring data. The likely date of local whitefly invasion is estimated, with a subsequent improvement of the model’s predictive accuracy. The method allows computation of the likely date of the first field incursion by the pest and demonstrates that the initial physiological age somewhat neglected in prior studies can improve the accuracy of model simulations. Given the increasing availability of monitoring data and models describing terrestrial arthropods, the integration of monitoring data and simulation models to improve model prediction and pioneer invasion date estimate will lead to better decision-making in pest management.

## 1. Introduction

Models have become indispensable tools used to integrate biological and ecological mechanisms. Models are used to identify the processes underlying the emergence of ecological phenomena, to quantify the relationships between abundance and environmental conditions, and to forecast the effects of changing environments on organismal populations [1–3]. Physiologically based models (PBMs) are one example; these models are commonly employed in agricultural and forestry contexts to characterize and eventually forecast insect pest outbreaks and their temporal progression [4,5]. The primary benefit of PBMs is the use of meteorological data and biological parameters including species-specific measurements obtained from laboratory experiments, to describe the evolution of the population abundance, in each life stage over time. These models are essential tools for improving the understanding and management of insect pest and disease vector dynamics, as they take into account the complex relationships and processes between life stages and the environment [4,5].

Various PBMs, including differential equation, cohort or individual-based models, have been developed and evaluated against *in situ* data from field population monitoring for a range of insect pests and crops. Examples include brown plant hopper, *Nilaparvata lugens* [6,7], rice leaf-folders, *Lepidoptera Pyralidae* [8], cotton bollworm, *Helicoverpa armigera* [9], fall armyworm, *Spodoptera frugiperda* [10] and the spotted wing drosophila, *Drosophila suzukii* [11]. To date, the practical implementation of insect pest models in decision support systems is still limited by two major issues [12]. The first is the implicit assumption that the initial insect population has not yet accumulated any effects of temperature, i.e. it has a physiological age of 'zero' at the beginning of the model simulations, neglecting the potential effects that environmental conditions may have acted on the organism prior to the start of monitoring. The second limitation is the absence of a robust method to calculate the initial number of immature individuals that were present when monitoring data show ‘no’ adult insects captured during the first trap visit. Hence, the size of the immature population (e.g. egg, larva, nymph or pupa) is rarely included in pest monitoring, although accurate model simulation in a near-future population requires acceptable initial conditions at all life stages. In other words, the estimation of initial conditions is quite challenging and one of the most critical elements of pest model applications [12,13]. The problem is exacerbated by the nonlinear dynamics of insect population growth and environmental heterogeneity under which populations evolve.

A PBM simulation will inevitably deviate from the real system trend if inappropriate initial conditions are used. The usual practice is to use a trial-and-error approach, adjusting the initial conditions until the simulation results show a pattern close to field data [9,11]. Bono-Rosselló *et al*. [12] used a more formal method involving the Kalman filter. However, although the relevance of the effect of initial physiological age in PBMs has been highlighted [13], the idea of using pest monitoring data to address the initial condition problem has not yet been fully explored. To the best of our knowledge, no study to date has provided a method that improves model accuracy by integrating the use of a mechanistic model and empirical field observation to estimate the size and physiological age of the cohort that initiated an outbreak in a given area of interest. To tackle this challenge, we propose utilizing a differential evolution algorithm [14], a popular tool in science and engineering for parameter estimation, in conjunction with field monitoring data and a weather-driven mechanistic model. The ultimate aims of this study are to estimate the initial physiological age and the population abundance at the time of the first sample, to improve the accuracy of predicting population changes over time and to investigate the timing of insect arrival at a field site by tracking back the physiological age of the initial cohort. The method we have developed works backwards from the initial sampling to the ‘real’ time zero of the infestation.

We introduce the model-data fusion method to estimate physiological age and cohort size in initial populations, with a case study involving whitefly, *Bemisia tabaci* (Hemiptera: Aleyrodidae) on cassava, the second most important staple food in sub-Saharan Africa [15]. This insect pest is a good candidate to test our framework because of the large literature on its biology and quantitative information that supports model development. Unfortunately, currently available simulation models do not explicitly consider the effect of weather drivers on whitefly population dynamics [16–18]. This crucial missing information in the literature, combined with the economic relevance of whitefly, provides an attractive contextual framework for model-data fusion.

## 2. Material and methods

This study introduces a generic technique that is potentially applicable to other terrestrial arthropods as well. To make the theoretical framework simpler to understand, we concentrate on the case study of *B. tabaci* for the purposes of illustration.

### 2.1. The target species

Whiteflies have direct effect as a pest of cassava [15], the insect is also a serious vector of cassava mosaic virus (CMV) [19] and cassava brown streak virus (CBSV) [20]. CMV is widespread through cassava-producing areas, with new strains providing additional threats of invasion [19]. CBSV is an epidemic pathogen that is spreading from East Africa, through Central and Southern Africa, towards the major cassava-cropping countries in West Africa [16]. The fecundity and maturation speed of whiteflies are highly dependent on temperature [21], thus variations in the weather affect the likelihood that the vector and hence the virus can spread to new planting areas. High population densities of *B. tabaci* can amplify the incidence of CBSV [15,22], rendering agricultural products unsuitable for market and consumption. It is, therefore, crucial to accurately predict their temperature-driven population dynamics over time.

### 2.2. Field trap population and temperature data

The data for the first physiological age estimate were originally collected at an experimental farm in Adiopoumé, 20 km west of Abidjan in the lowland rainforest zone of Côte d'Ivoire, West Africa. The farm consisted of 1 ha plots where a mixture of crops, including cassava, were cultivated [23]. Separate trials, planted in the 1988, 1989 and 1990 growing seasons, were conducted. Adult and nymph whiteflies were monitored twice per week by direct sampling of infected plants [23]. The experimental layout was composed of 49 blocks (7 by 7) with 2 m spacing between blocks, each containing 100 plants spaced 1 × 1 m apart (4900 plants in total). Separate trials, planted in November/December and monitored for a minimum of four months, were conducted in 1988, 1989 and 1990. The trials were planted at the beginning of the dry season. Each year, the 0.75 ha experimental field was oriented such that the dominant southwest winds crossed the upwind field border at right angles [23]. Adult *B. tabaci* monitoring began two to three weeks after planting, when the canopy of the newly planted crop was sufficiently developed and more susceptible to infestations by adults. Adults were monitored twice per week, by random visual inspections on 10 plants per block. One of the weekly counts instead followed a transects scheme along the blocks, in which the same 490 plants were monitored on each occasion. For the other count, two randomly selected plants per block plus their four nearest neighbours were chosen. Data were obtained by counting the adults on the undersurfaces of the top five leaves of one growing apex per plant. Counts were carried out mid- to late morning when the wind speed had increased, conditions under which *B. tabaci* is reluctant to fly. *Bemisia tabaci* nymph monitoring began approximately five weeks after planting and thereafter continued weekly using a hand lens. Specimens were counted on one plant per block and the monitoring was restricted to the central three folioles of leaves 11−20 from the growing tip. Temperature data at Adiopoumé for the trapping periods in 1988−1990 were obtained from the National Aeronautics and Space Administration (NASA) Langley Research Center (LaRC) Prediction of Worldwide Energy Resource (POWER) [24].

### 2.3. Model overview

**Figure 1.**
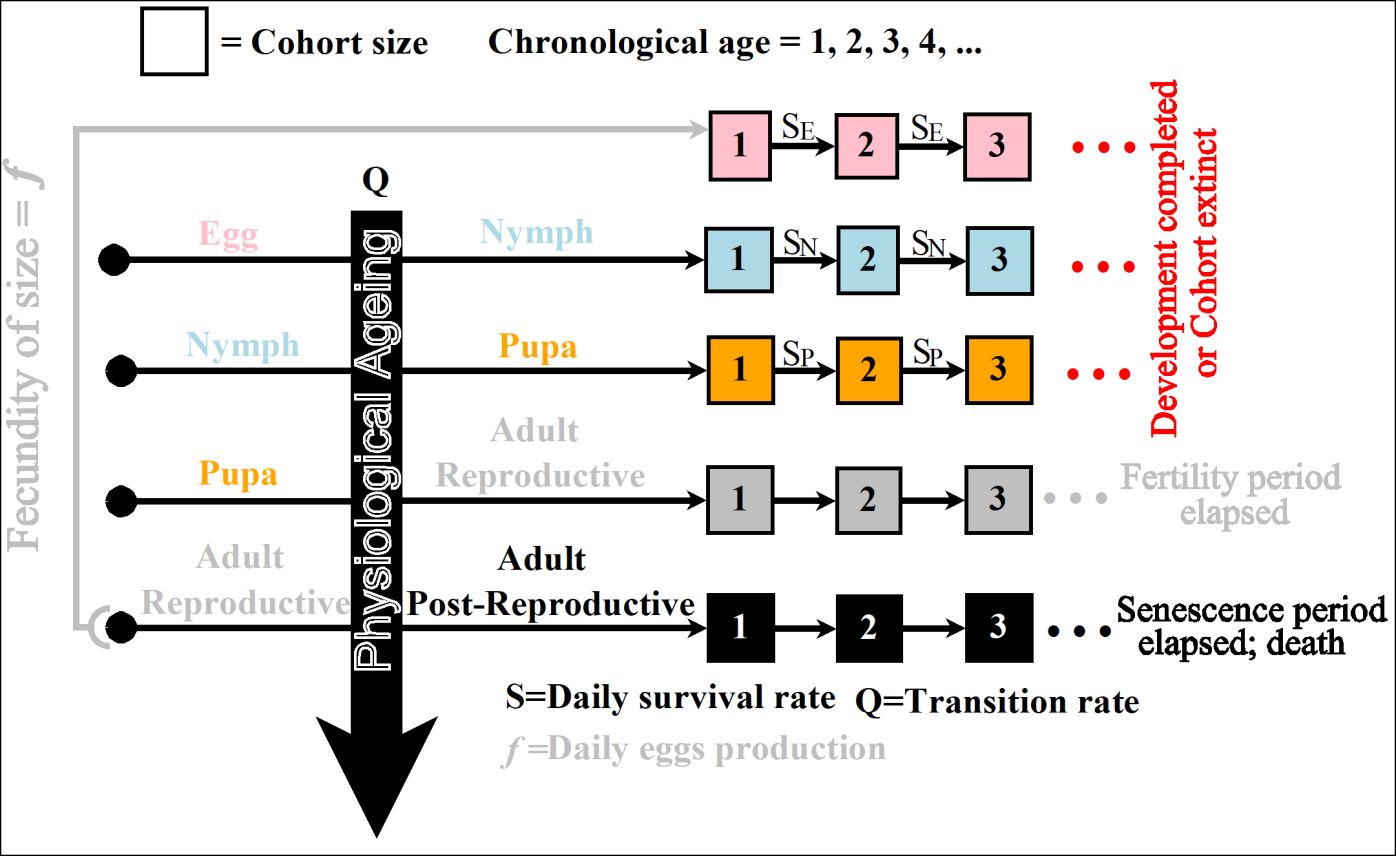
This schematic representation illustrates the overall modelling process of whitefly population dynamics. The process starts with newly emerged females that are reproductive and produce a total number of eggs (f) that compose the cohort of size f. A fraction of these eggs moves to the nymph stage, then the ‘pupa’ stage, and finally the mature life stage, following a transition rate Q. The size of the cohort of eggs, nymphs and ‘pupae’ undergoes a daily reduction, dependent on the survival rates $S_{E}$, $S_{N}$ and $S_{P}$, respectively. The adults become post-reproductive at a rate Q after the end of the fertility period, interrupting egg production and die when the senescence period is completed. Cohorts that are completely extinct or senesced are removed from computations.

Extensive studies of thermal biology and the population ecology of *B. tabaci* provide a wealth of quantitative data for model applications [18,21,23]. Using monitoring data [23], as well as information on the physiological effects of temperature [21], we developed a detailed PBM of *B. tabaci* population. We included temperature-driven demographic variables that affect population variation at each life stage, combined with on-site pest monitoring trap data to estimate initial abundance and physiological age of cohorts at each immature life stage. The ad hoc script code developed for simulations is publicly available [25] and written in Intel^®^ Fortran Compiler [26] through Microsoft Visual Studio [27] to facilitate more rapid calculation. The statistical analysis and output data were graphically represented by using the software R [28] (please refer to figure 1 and appendix for an overview of the model structure and equation formulation).

### 2.4. Simulation initialization

**Figure 2.**
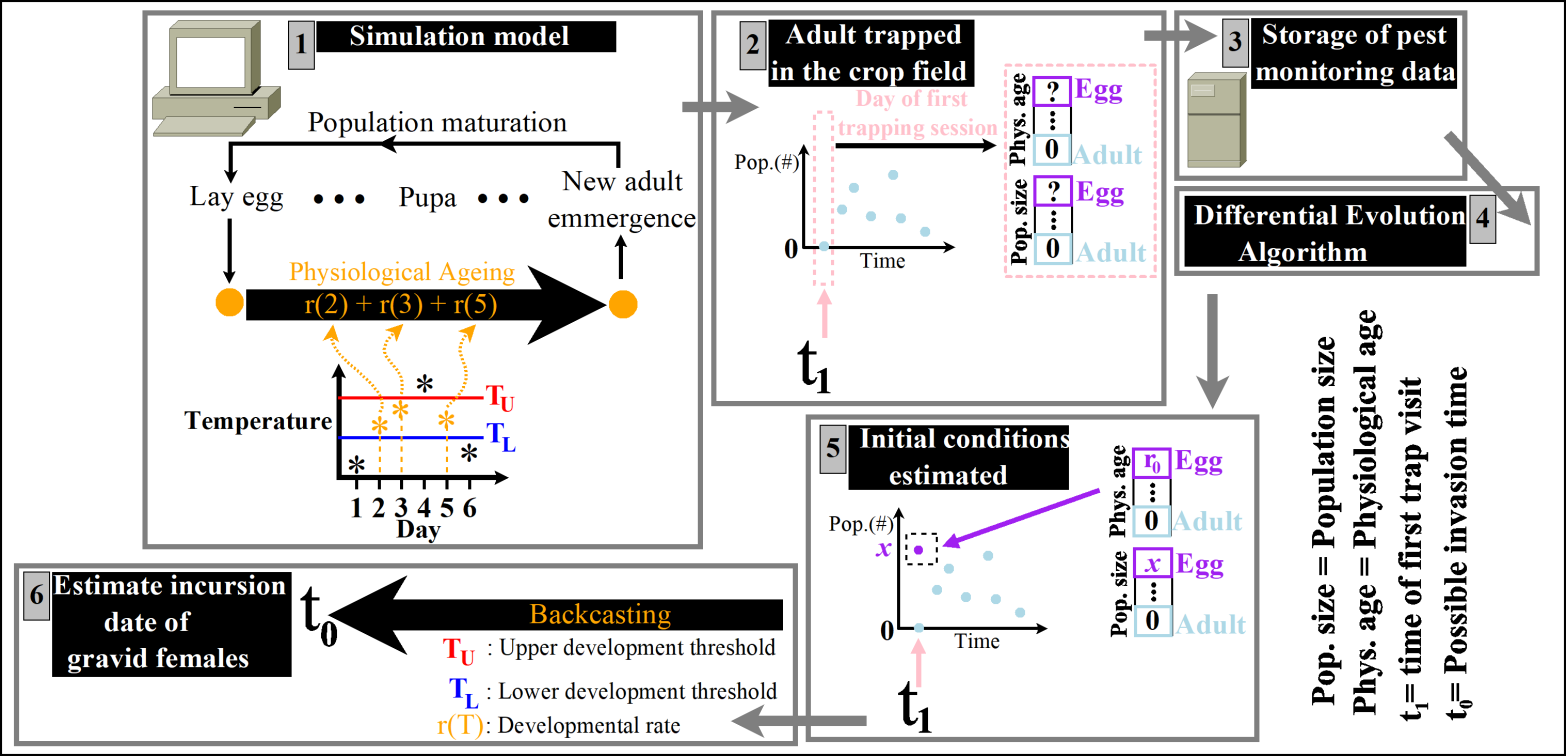
Illustration of the recalibration and simulation process (1). The simulation model containing the key mechanisms involved during the temperature-driven life cycle of the insect is constructed (2). The adult trap data are identified for use in parameter estimation (3,4). Combining the optimization and simulation models, the initial population size and physiological age are estimated. Once the initial conditions are estimated (5), the back-casting algorithm is based on the ‘inversion’ of the forward physiological ageing process (refer to the text and figure 3 for additional details) and is executed to estimate the calendar date of possible first field infestation (6).

The simulation model starts by specifying the size of the initial cohorts. The physiological ages for each of the initial cohorts are specified as well. The model was designed to track the increase of physiological age and the transition of *B. tabaci* through each development stage. The population size $P_{i}$ of each cohort i (i = egg, nymph, ‘pupa’ or adult) is tracked, with additional allowance for natural mortality and egg production by fertile females (see figures 1 and 2). The physiological age and cohort size are tracked and updated throughout each simulation.

**Figure 3.**
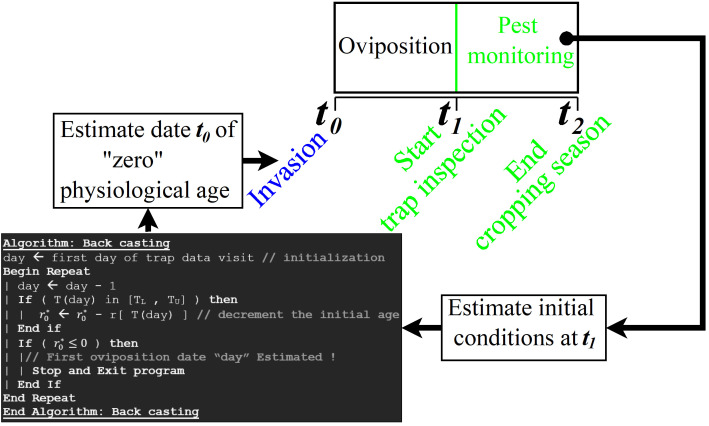
Schematic of the sequence of events during pest monitoring and the algorithm used to estimate the likely invasion date ($t_{0}$). The function $r\left[T\right]$ is the temperature-dependent developmental rate, the other variables are defined in the text.

### 2.5. Physiological ageing and development

The ontogenic cycle of *B. tabaci* from egg to an adult is contingent on the temperature conditions it encounters during its immature life stages [21]. In this model, we subdivide *B. tabaci* into egg, nymph, ‘pupa’ and adults (reproductive and post-reproductive). In strict entomological terms, *B. tabaci* is a hemimetabolous insect, meaning it does not undergo a true pupal stage but instead transitions directly from the final instar to the adult form. To be consistent with the literature on *B. tabaci* and from a modelling perspective, ‘pupa’ refers to the intermediate stage between nymph and adult [15,21,23]. We model the progression from egg to adult using the principle of thermal rate accumulation [29], as illustrated in figures 1 and 2.

The development rate represents the progress of a given life stage per time unit (i.e. per hour, per day, ..., etc…). The physiological age accumulation over time ranges from 0 to 1 (i.e. evolution from 0 to 100% accumulated developmental phase). When it is equal to 1 (i.e. 100%), the stage-development is completed. The cumulative developmental progress over time (t) or physiological age can be computed as described by Chuine & Régnière [29],


$$Q_{i\to j}=\int r_{i\to j}\left(T\left(t\right)\right)dt.$$

The subscripts i→j stand for egg to nymph, nymph to ‘pupa’, ‘pupa’ to adult. The function $r_{i\to j}\left(T\right)$ is the temperature-dependent development rate from the current (i) to the next life stage (j) (see appendix). The integral $Q_{i\to j}$ can be approximated with a discrete-time step (Δt) summation when, for example, average daily temperature is used [29],


$$Q_{i\to j}≅r_{0}^{i}+\sum_{day=1}^{day=n}r_{i\to j}\left(T_{day}\right)\times \Delta t,$$

where $T_{day}$ is the average environmental temperature at the day (n), and the time-step Δt=1day. The physiological age ($Q_{i\to j}$) is updated only if the mean daily temperature falls in the permissive range [$T_{L}^{i}$, $T_{U}^{i}$], where $T_{L}^{i}$ and $T_{U}^{i}$ (i = egg, nymph or ‘pupa’) are the lower and upper developmental thresholds, respectively (see figure 2 and appendix). The additional parameter $r_{0}^{i}$ corresponds to be the initial physiological age (positive and lower than 1) at the time of the first trap visit for the immature life stage i. It can also be considered as a way of indicating how far the initial cohort has progressed in its development at the time of the first field observations. The cohort turns into a next-life state once $Q_{i\to j}=1$.

### 2.6. Survival

The daily survival rate is computed at each life stage i using the equation $S_{i}={\left(1-m_{i}(T_{day})\right)}^{r_{i\to j}(T_{day})}$ [30–32]. The function $m_{i}$ is the immature temperature-dependent mortality rate (please refer to the appendix for a detailed description). The lower and upper lethal temperatures for whiteflies are LLT = 4°C and ULT = 36°C, respectively [21]. Outside the permissive temperature range [LLT , ULT], survival is $S_{i}=0$. Once the survival rate during the current day is estimated, the population density ($P_{i}$) is updated to $S_{i}P_{i}$.

### 2.7. Fecundity

Newly emerged adults are assumed to be reproductive. The total number of eggs produced by females per day generates a new cohort of eggs with size,


$$f=S_{R}\phi \left(T_{day}\right)\psi \left(T_{day}\right)N_{A}.$$

Although it can slightly vary with temperature [21], the sex ratio $S_{R}$ is assumed to be 50% for the sake of simplicity. The oviposition rate, $\phi (T_{day})$ (in day^−1^), is the fraction of eggs from the optimum oviposition capacity $\psi (T_{day})$. The parameter $\psi (T_{day})$ is the maximum number of eggs a female can produce in her lifetime. The variable $N_{A}$ represents the total number of reproductive adults. The model tracks the evolution of each new cohort of size f separately (figure 1). Egg production occurs within the temperature range [4°C, 36°C] [21]. The fecundity period of a female cohort is elapsed once ∫ϕ(T(t))dt=1. After completion of the fecundity period, the adult female enters the post-reproductive life stage and is unable to lay eggs any more. The post-productive female dies after a completed accumulation of the senescence rate $\varphi (T_{day})$, i.e. ∫φ(T(t))dt=1 (the functions φ, ϕ and ψ are provided in the appendix).

### 2.8. Density and host crop effects

To capture the effects of composite sources of predation/parasitism mortality, intraspecific interactions and density, and to keep the dynamics within reasonable bounds, density-dependent survival is included in the model as an additional source of natural mortality. Density-dependent survival is inversely proportional to the number of nymphs ($N_{n}$) [18],


$$s_{D}=\frac{1}{1+KN_{n}}.$$

In the nymphal stage, whitefly usually settles on the same host plant on which they emerged and are affected by the ageing of the cassava crop, which is detrimental to their survival [15]. The density-dependent survival, $s_{D}$ is multiplied by the function $C_{S}$, which represents the decline in crop suitability over time [18]. The parameter K (strength of density dependence) is estimated from field population dynamics data using the procedure described in the following sections.

### 2.9. Parameter estimation and backward projection

Following the procedure shown in figures 2 and 3, the simulation model and trap data are conjointly used to estimate the initial (physiological age and density) using the difference evolution (DE) algorithm [14]. The algorithm is initialized by providing a plausible range for each value to estimate: (i) initial cohort size of egg, nymph and ‘pupa’ within $\left[0,10^{6}\right]$; (ii) $r_{0}^{i}\in \left[0.0,0.99\right]$ (i= egg, nymph or ‘pupa’), and (iii) $K\in \left[10^{-5},10^{2}\right]$. With a succession of initial parameter mutation, crossing and selection processes (see electronic supplementary material) [14], and having the field sample size Ω, the DE algorithm iteratively optimizes the residual error sum of square (RSS) for the difference between observed ($O_{k}$) and simulated ($S_{k}$) at field sampled at day = k. Combining the total of Ω observations and simulations data,


$$\text{RSS}=\sum_{k=1}^{\Omega }{\left[O_{k}-S_{k}\right]}^{2}.$$

The DE optimization stops when the objective (or cost) function (RSS*)* is optimally minimized [14].

### 2.10. Model evaluation

The modelled output values are plotted versus the corresponding measured data and analysed by a set of statistical properties (*F*-statistics *p*‐value, *R*^2^ and Akaike information criterion (AIC)) for the observed data/model data regression line coefficients (slope and intercept) [33]. This provides a way of checking whether the regression line is statistically similar to the 1 : 1 line (perfect accordance between simulation and data) [33]. Once the (optimal) initial physiological age ($r_{0}^{,}$) value is estimated, the pseudo-code below for back-casting (see figure 3) is executed and the likely date of first egg oviposition is evaluated.

## 3. Results

**Figure 4.**
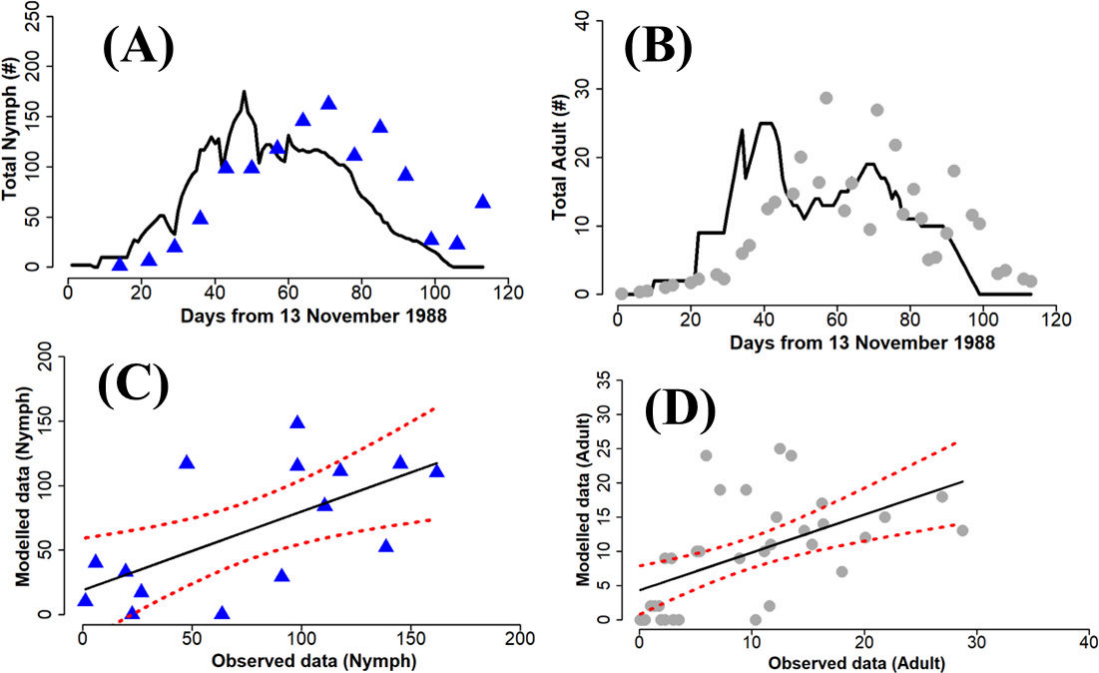
Comparison of model output with field data for nymph and adult whitefly during the 1988 field monitoring. Linear regression Nymph: $R^{2}$=0.41, *p* = 0.01, AIC = 157.43. Adult: $R^{2}$ = 0.31, *p*l < 0.001, AIC = 234.79. The blue triangles (A) and the grey circles (B), respectively, represent the nymph and adult data collected in the field, while the solid black line is the simulation result. The dashed red lines in the linear regression (C) and (D) indicate the 95% confidence intervals.

**Figure 5.**
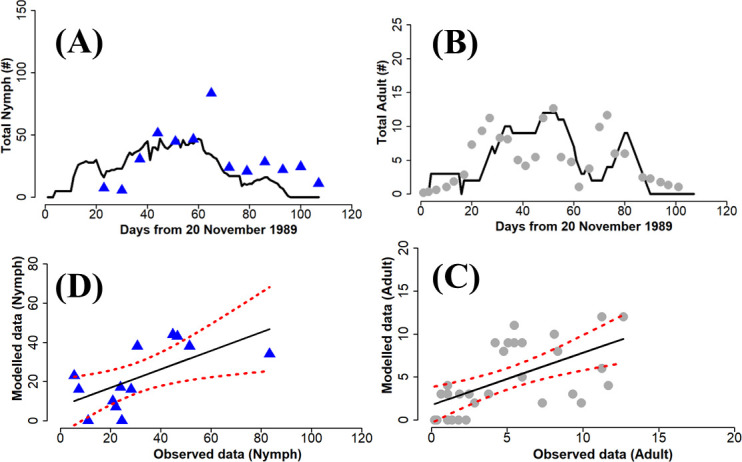
Comparison of model output with field data for nymph and adult whitefly during the 1989 field monitoring. Linear regression Nymph: $R^{2}$= 0.40, *p* = 0.02, AIC = 106.96. Adult: $R^{2}$= 0.35, *p* < 0.001, AIC = 155.08. The blue triangles (A) and the grey circles (B), respectively, represent the nymph and adult data collected in the field while the solid black line is the simulation result. The dashed red lines in the linear regression (C) and (D) indicate the 95% confidence intervals.

**Figure 6.**
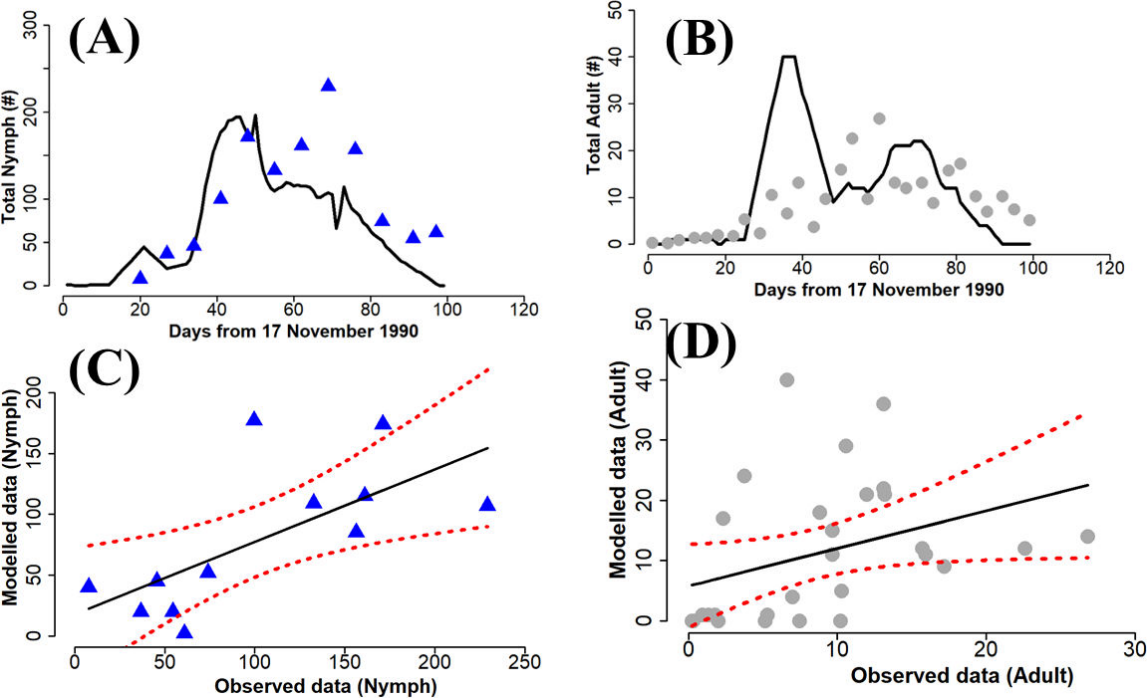
Comparison of model output with field data for nymph and adult whitefly during the 1990 field monitoring. Linear regression Nymph: $R^{2}$= 0.46, *p* = 0.01, AIC = 129.33. Adult: $R^{2}$= 0.13, *p* = 0.05, AIC = 224.80. The blue triangles (A) and the grey circles (B), respectively, represent the nymph and adult data collected in the field while the solid black line is the simulation result. The dashed red lines in the linear regression (C) and (D) indicate the 95% confidence intervals.

The performance of the parametrized model compared with field data from Fishpool *et al*. [23] is shown in figures 4–6. The simulation trajectories tracked the trends in the dynamics observed in the field data, including the date of maximum peak in the population. Overall, the predictions for the nymph life stage are more accurate than for the adult life stage.

The initial abundance and physiological age (cf. $t_{1}$ in figure 3) of the egg, nymph and ‘pupa’ cohort are provided in table 1. The backward iteration (see figure 3) estimated the day of physiological age ‘0’, i.e. when the possible first oviposition is likely to have occurred (cf. $t_{0}$ in figure 3), for the eggs on 22 October 1988, 30 October 1989 and 30 October 1990, i.e. when the first oviposition is estimated to have occurred. The initial conditions calculated with physiological age fixed at zero (table 1) have been carefully examined, and we found that they are often larger than those with non-zero initial physiological age.

**Table 1.** Estimated initial conditions (cf. at $t_{1}$ in figure 3); pop. size = population size, phys. age = physiological age. The figures in parentheses stand for initial physiological age fixed at zero.

initial cohorts at $t_{1}$	immature life stages			season
	egg	nymph	‘pupae’	
pop. size	10 (5)	2 (2)	1(0)	1988
phys. age	0	0.49	0.55	
pop. size	5 (672)	0 (0)	4 (7)	1989
phys. age	0.62	0	0.32	
pop. size	1 (1)	2 (0)	0 (2)	1990
phys. age	0.17	0.87	0	

For comparison purposes, the initial physiological age is set to 0, as if there was no prior effect of temperature. The performance of the simulation model and its accuracy were reduced, when the physiological age at initial survey time was not taken into account during the calculation process (table 2), highlighting the importance of physiological age in estimating the initial conditions. A closer look at the simulations with and without allowance for physiological age at the time of the initial survey (see electronic supplementary material, figures S1–S3) shows close agreement between the model and corrected data for physiological age during two out of the three years.

**Table 2.** Comparison of the model performance when the initial physiological age (cf. at $t_{1}$ in figure 3) is fixed at 0 and when it is calibrated.

performance	season	initial pop. size estimated and initial physiological age fixed to zero	initial pop. size +age estimated
RSS	1988	32748.82	31096.80
	1989	5428.27	4681.40
	1990	40854.91	38800.54

## 4. Discussion and conclusion

### 4.1. Model and monitoring data fusion in insect pest modelling

This work introduced a new method for accurately estimating the initial conditions of a PBM using field monitoring data. While it is acknowledged that species-specific limitations, model assumptions and data availability may affect the applicability and transferability of the approach, the successful testing of the methodology on the whitefly *B. tabasi* can serve as an inspirational case study for adaptation to other crops and insect pests.

Discrepancies between the model and data may be due to the fact that adult specimens could fly away if disturbed during the sampling process. Conversely, nymphs remain on the plant where they have emerged until they reach maturity. Consequently, it can be hypothesized that the model is more accurate in describing nymph than adult populations because of *in situ* data sampling bias. An additional limitation of the model is the lack of terms that account for mortality caused by abiotic factors (e.g. rain, relative humidity) and biotic factors (e.g. predators, parasitoids, disease and competitors).

Fixing the initial physiological age at zero, expands the period during which death can occur (modelled by the daily death rate) leading to an ‘artificially’ reduced cohort size. Some authors have overcome the problem by assuming a relatively large initial population size [9,12,34], thus ensuring that a higher proportion of the initial population reaches adult or advanced preimaginal life stage. We adopt a different approach in which the initial physiological age ≠ 0, thus assuming the initial population is already ‘older’ in terms of physiological age. In this case, even a small proportion of the initial cohorts have a higher chance of reaching maturity.

Our results show that there is a real improvement in the agreement between simulations and observational data when the initial conditions at the time of first survey are rigorously estimated instead of the conventional approach of arbitrarily selecting initial conditions until a simulation trajectory aligns with observational data [9,12,34]. The latter trial-and-error can lead to an overestimation of the first cohort size. Our approach, therefore, represents a contribution in the field of simulation modelling of insect pest population dynamics.

Our methodology is aligned with the current trend of integrating model simulations and field data to enhance the accuracy and reliability of simulation outcomes. For instance, Rossini *et al*. [34] estimated the parameters of temperature-dependent developmental rates of the immature life stages using genetic algorithms and PBMs, with the spotted wing drosophila (SWD) serving as a case study. Bono-Rosselló *et al*. [12] employed Kalman filter techniques to refine the model projections by integrating monitoring data, thereby demonstrating that the model’s dependence on initial conditions is markedly diminished in the case of SWD. Our work represents a comprehensive addition to this framework [12,34], offering a precise methodology for estimating initial conditions of the physiological age of the populations at the time of the first survey.

A degree-day model and trap data have also recently been combined to estimate the emergence date of adult pests in the field [35,36]. However, unlike the current study, the models of Rincon *et al*. [35] and Sasaki *et al*. [36] are not mechanistic, in terms of modelling the effects of fluctuating temperature on egg, nymph, pupa and adult dynamics (cf. figures 1 and 2), but instead, consider a linear temperature-dependent development rate.

### 4.2. The case of *B. tabaci*

While other examples of PBMs have been developed for whitefles, they neither consider the entirety of the life cycle nor conduct a comprehensive comparison with population monitoring data at multiple life stages [37,38]. Moreover, previous models were not oriented towards cassava-specific whiteflies, nor to sub-Saharan Africa (SSA)–East and Southern Africa (ESA) *B. tabaci* feeding on cassava crops [21]. The present study introduces a novel temperature-driven mechanistic model that describes the stage-population dynamics of the SSA-ESA whitefly, in contrast to species distribution modelling [17,39] or, fixed and temperature-independent biodemographic rate models [18].

### 4.3. Implication for pest management

The use of traps is a common practice in insect monitoring, as well as in the formulation and implementation of pest management strategies and control actions [40–43]. Typically, however, traps track only the population dynamics of one stage (usually the adult) or one sex (in case of sexual pheromone lures) [40–43], providing no information on the preimaginal stages. Pest management is, therefore, constrained if it is based solely on monitoring data without additional analyses when coupled with population models. Effective control strategies ought to be planned on the basis of future trends in infestation, whereas monitoring alone provides only a snapshot of the present and offers no insight into future developments. Control strategies can target several preimaginal stages, and an empirical estimation based on data from a single stage is challenging and unreliable. The type of physiologically based model introduced here offers a means to predict future spread that takes account of individual stage dynamics.

Models alone do not necessarily guarantee reliable predictions without input data from the field. This is why the combination of data from monitoring and *in silico* elaboration can provide a more comprehensive and accurate overall picture [12,34–36,44]. Guimapi *et al*. [44] back-calculated the date of early field infestation of fall armyworm using range-censored data from egg and larva stages collected from maize plants at the young leaf development stage. In contrast to the approach taken by Guimapi *et al*. [44], the present study applied a more formal methodology, integrating a mechanistic population model with monitoring data to estimate the most likely date of initial field incursion.

### 4.4. Future work

The methodology introduced in the present study allows the estimation of the date and magnitude of the initial field infestation. Further work involving a spatially explicit extension of the present model, could, in principle, be used to predict dispersal distances and to inform the optimal timing and location for trap placements.

In addition to their use in the context of agricultural pests, the findings of the present work may also have potential applications in forensic entomology. This is with a view to determining the time of death of an animal using insects found on the carcass [45]. We believe that by adapting the methodology employed in this study, a more precise prediction of the initial oviposition date of the female insect in the carcass could be achieved.

## Data Availability

All simulations and numerical calculations were performed with Intel® Fortran. The custom code that supports the findings of this study is available at online Zenodo repository [25]. Supplementary material is available online [46].

## Appendix A.

See table 3.

**Table 3.** Temperature-dependent bio-demographic functions for the Whitefly. The variable 𝑇 indicates the temperature in (°C).

variable	definition (unit)	mathematical expression	parameters	data source
$r_{1\to 2}$	egg developmental rate (day^-1^)	$\frac{a(T-d)}{1+b^{\left(T-c\right)}}$	$\begin{array}{l} a=0.008426\\ c=38.02\\ d=10.05\\ b=2.119 \end{array}$	[21]
$r_{2\to 3}$ $r_{3\to 4}$	nymph developmental rate (day^-1^) ‘pupa’ developmental rate (day^-1^)	$e^{\rho T}-e^{(\rho T-\frac{k-T}{\delta })}+\lambda$	$\begin{array}{l} \rho_{2\to 3}=0.008053\\ k_{2\to 3}=46.837933\\ \delta_{2\to 3}=5.894955\\ \lambda_{2\to 3}=-1.105855\\ \rho_{3\to 4}=0.010529\\ k_{3\to 4}=38.647402\\ \delta_{3\to 4}=2.057230\\ \lambda_{3\to 4}=-1.046143 \end{array}$	[21]
$m_{1}$	egg mortality (%)	$1-\frac{1}{e^{((1+e^{(\frac{-(T-T_{opt})}{B_{l}})})(1+e^{(\frac{-(T_{opt}-T)}{B_{h}})})h)}}$	$\begin{array}{l} b_{l}=6.54777\\ b_{h}=0.91766\\ h=0.04061\\ T_{opt}=32.67099 \end{array}$	[21]
$m_{2}$	nymph mortality (%)	$1-\frac{1}{e^{((1+e^{(\frac{-(T-T_{opt})}{B_{l}})})(1+e^{(\frac{-(T_{opt}-T)}{B_{h}})})h)}}$	$\begin{array}{l} b_{l}=3.87681\\ b_{h}=2.20412\\ h=0.09057\\ T_{opt}=26.59778 \end{array}$	[21]
$m_{3}$	‘pupa’ mortality (%)	$e^{\left(b_{1}+b_{2}T+b_{3}T^{2}\right)}$	$\begin{array}{l} b_{1}=10.7852566\\ b_{2}=-1.2365342\\ b_{3}=0.0260262 \end{array}$	[21]
φ	senescence rate (day^-1^)	$\frac{c_{1}}{1+e^{\left(k_{1}+k_{2}T\right)}}-\frac{c_{2}}{1+e^{\left[k_{1}+k_{2}(2T_{opt}-T)\right]}}$	$\begin{array}{l} c_{1}=0.114\\ c_{2}=0.1248\\ k_{1}=-6.0039\\ k_{2}=0.331102\\ T_{opt}=22.43182 \end{array}$	[21]
ψ	total oviposition (# eggs/female/lifetime)	$e^{\left(b_{1}+b_{2}T+\frac{b_{3}}{T}\right)}$	$\begin{array}{l} b_{1}=18.9757\\ b_{2}=-0.2894\\ b_{3}=-174.1446 \end{array}$	[21]
ϕ	oviposition rate (day^-1^)	$\frac{c_{1}}{1+e^{\left(k_{1}+k_{2}T\right)}}-\frac{c_{2}}{1+e^{\left[k_{1}+k_{2}(2T_{opt}-T)\right]}}$	$\begin{array}{l} c_{1}=0.075487\\ c_{2}=0.176870\\ k_{1}=-7.606472\\ k_{2}=0.360645\\ T_{opt}=24.359020 \end{array}$	[21]
$T_{U}^{i}$ $T_{L}^{i}$	upper development threshold (°C) lower development threshold (°C)	—	$\begin{array}{l} T_{U}^{1}=36.0\\ T_{U}^{2}=36.0\\ T_{U}^{3}=36.0\\ T_{L}^{1}=10.18476\\ T_{L}^{2}=11.78421\\ T_{L}^{3}=5.228402 \end{array}$	[21]
$C_{s}$	crop suitability (no dimension)	$\frac{e^{\left[a_{1}-a_{2}\text{log(day − 1)}\right]}}{a_{3}}\text{if day}<\text{80 and 1.0 otherwise}$	$\begin{array}{l} a_{1}=15.026\\ a_{2}=2.389\\ a_{3}=100.0 \end{array}$	[18]
	lower lethal threshold (°C) upper lethal threshold (°C)	—	$\begin{array}{l} LLT=4.0\\ ULT=36.0 \end{array}$	[21]

## Appendix B. Model formulation

The model describes the dynamics of the complete life cycle of the whitefly based on daily temperature. We assume that there is neither emigration nor immigration, which in a field context, can be interpreted as an *equilibrium* between the outcoming and incoming individuals from neighbouring fields. The life cycle of the *B. tabaci* can be divided into five life stages: egg, nymph, ‘pupa’, reproductive adult and post-reproductive adult. Individuals at each stage are grouped into cohorts, and age of each cohort is given by tracing the number of days since the transitions to the current life stage (figure 1). Let E(t,k) be the number of eggs of age k at time t, N(t,k), P(t,k), RA(t,k) and PRA(t,k) be that for nymph, ‘pupae’, reproductive adults and post-reproductive adults, respectively. The population dynamics develop in the sequence E→N→P→RA→PRA→∎ and it is described by stage-specific survivorship and fecundity. The symbol ‘∎’ stands for an extinct (or death) cohort. The stage-specific survival rate, given by $S_{E}$, $S_{N}$, $S_{P}$, are rates from (chronological) age k to k+1 at each developmental stage of egg, larva, ‘pupa’ and adult, respectively. The nymphs undergo an additional mortality proportional to their density with survival rate $S_{D}$. The fecundity, f, is assumed to be constant with age. We represent the pest abundance by the number of females and males but the egg quantity is proportional to the number of reproductive females (see the main text for more details). The cohort size in the following day is the current cohort size multiplied by survival rate at time t. The survival rate varies over time depending on the temperature, and the population size is large enough to neglect demographic stochasticity. Once the model is initialized (see the main text), the population dynamics are formulated as

$$E\left(t+1,1\right)=f\sum_{k=1}^{\Omega_{\text{RA}}}\text{RA}\left(t,k\right)$$

$$E\left(t+1,k+1\right)=S_{E}\left[1-Q_{E\to N}\left(t,k\right)\right]E\left(t,k\right)\;for\;k=1,2,\cdots ,\Omega_{E}-1$$

$$N\left(t+1,1\right)=\sum_{k=1}^{\Omega_{E}}Q_{E\to N}\left(t,k\right)E\left(t,k\right)$$

$$N\left(t+1,k+1\right)=S_{N}\left[1-Q_{N\to P}\left(t,k\right)\right]N\left(t,k\right)+S_{D}N\left(t,k\right)\;for\;k=1,2,\cdots ,\Omega_{N}-1$$

$$P\left(t+1,1\right)=\sum_{k=1}^{\Omega_{N}}Q_{N\to P}\left(t,k\right)N\left(t,k\right)$$

$$P\left(t+1,k+1\right)=S_{P}\left[1-Q_{P\to \text{RA}}\left(t,k\right)\right]P\left(t,k\right)\;for\;k=1,2,\cdots ,\Omega_{P}-1$$

$$\text{RA}\left(t+1,1\right)=\sum_{k=1}^{\Omega_{P}}Q_{P\to \text{RA}}\left(t,k\right)P\left(t,k\right)$$

$$\text{RA}\left(t+1,k+1\right)=\left[1-Q_{\text{RA}\to \text{PRA}}\left(t,k\right)\right]\text{RA}\left(t,k\right)\;for\;k=1,2,\cdots ,\Omega_{\text{RA}}-1$$

$$\text{PRA}\left(t+1,1\right)=\sum_{k=1}^{\Omega_{\text{RA}}}Q_{\text{RA}\to \text{PRA}}\left(t,k\right)\text{RA}\left(t,k\right)$$

$$\text{PRA}\left(t+1,k+1\right)=\left[1-Q_{\text{PRA}\to ∎}\left(t,k\right)\right]\text{PRA}\left(t,k\right)\;for\;k=1,2,\cdots ,\Omega_{\text{PRA}}-1,$$

where $\Omega_{E}$, $\Omega_{N}$, $\Omega_{P}$, $\Omega_{\text{RA}}$ and $\Omega_{\text{PRA}}$ are the lifespan limits of eggs, nymph, ‘pupa’, reproductive adults and post-reproductive adults, respectively. The variable $Q_{i\to j}(t,k)$ is the probability that cohort at stage i of age k transfer into the stage j at time t. The particular case $Q_{\text{PRA}\to ∎}$ represents the removal of the cohort from the simulation, since no life stage exists beyond PRA. The law of effective cumulative temperature determines these transition probabilities.

Transition from the current to the next stage is assumed to occur when the thermal rate $r_{i\to j}$ accumulated ($\int r_{i\to j}$) is equal to 1; which is called ‘the law of effective cumulative temperature’ [29]. Therefore, the temperature (T) to which the pest population is exposed critically influences the duration time of each life stage. The probability of the development of individuals at stage i (i represents either eggs, nymph, ‘pupae’, reproductive or post-reproductive adults) of age k to the next stage is described by

$$Q_{i\to j}\left(t,k\right)=\left\{ \begin{array}{l} 1\;\;if\;\;\sum_{l=1}^{k}r_{i\to j}\left(T\left(l\right)\right)\geq 1\\ 0\;\;if\;\;\sum_{l=1}^{k}r_{i\to j}\left(T\left(l\right)\right)<1. \end{array}\right.$$
